# Optical neural networks with intensity‑based projection layers as effective nonlinear activations

**DOI:** 10.1038/s41598-025-30632-y

**Published:** 2025-12-09

**Authors:** Jiawei Xi, Randy Stefan Tanuwijaya, Tan Li, Jensen Li

**Affiliations:** 1 Department of Physics, The Hong Kong University of Science and Technology, Clear Water Bay, Kowloon, Hong Kong China; 2 Centre for Advances in Reliability and Safety (CAiRS), Hong Kong Science Park, Pak Shek Kok, NT, Hong Kong China; 3 Department of Engineering, University of Exeter, Exeter, United Kingdom

**Keywords:** Optical physics, Applied physics

## Abstract

Optical neural networks (ONNs) offer significant advantages in speed and energy efficiency by exploiting the parallelism of light. A key challenge in their design is the implementation of nonlinear activation functions using physically realizable operations. In this work, we propose the use of optical intensity measurements—as projections from complex or quaternion-valued fields to real-valued magnitudes together with amplitude or phase encoding to next layer—as effective alternatives to traditional nonlinear activation functions in neural networks. Through systematic evaluations across image classification, image reconstruction, and physical parameter inference, we validate the effectiveness and robustness of projection layers as nonlinear activation mechanisms. The approach extends naturally to quaternion-valued representations, capturing multi-dimensional features such as amplitude, phase, and polarization. We further assess performance under noisy conditions and demonstrate its utility in a β-VAE framework for learning interpretable physical parameters. This projection-based approach not only leverages the advantages of optical computing but also establishes a scalable and hardware-efficient foundation for deep ONNs with broad applicability in image recognition, signal processing, and scientific inference.

**Supplementary Information:**

The online version contains supplementary material available at 10.1038/s41598-025-30632-y.

## Introduction

Recent advancements in optical neural networks (ONNs) have opened new avenues for harnessing the inherent parallelism and high-speed processing capabilities of light-based computation, particularly in tasks such as image classification and computational imaging using the deep diffractive neural networks (D^2^NN)^1–7^. Unlike traditional neural networks, which rely on electronic circuits and are constrained by power consumption and processing bottlenecks, ONNs encode inputs as optical fields and perform computation using components such as spatial light modulators (SLMs), digital micromirror devices (DMDs) and linear diffraction layers^8–14^. The natural parallelism of light propagation and diffraction, combined with multiplexing techniques that exploit physical degrees of freedom—including wavelength, polarization, spatial mode, and time—enables ONNs to achieve efficient, low-latency operations with significant energy savings^15–17^.

Furthermore, the incorporation of nonlinear activation functions—a fundamental component of deep learning—has been reinterpreted in ONNs through various physical phenomena, including the Kerr effect, free-carrier dispersion, quantum interference, and multiple scattering^18–26^. These mechanisms offer natural pathways for introducing nonlinearity into optical systems, enabling the complex transformations required for effective data representation and decision-making. In addition, the measurement of light intensity, following the propagation of complex-valued optical fields, can serve as an implicit nonlinear activation function^11,27,28^. This process acts as a projection layer, mapping complex amplitudes to their intensity, thereby extracting modulus-based features from the underlying complex representation. Such a projection-based approach not only circumvents many of the practical challenges associated with realizing optical nonlinearity^10,22,26^, but also significantly simplifies the physical implementation of ONNs. While prior works have acknowledged this mechanism, a systematic investigation of its potential as a standalone method for feature extraction and nonlinear transformation remains lacking.

To address this gap, we propose a systematic approach that employs complex projection, together with an encoding scheme, as an effective and physically realizable nonlinear activation function within neural networks. By projecting complex-valued representations—corresponding to electric fields—onto their intensity measurements, the network achieves dimensionality reduction while preserving the capacity for complex feature transformations. We further extend this concept to higher-dimensional representations by introducing quaternion-valued projections, which generalize the approach from two to four components prior to projection. The resulting projection layers are evaluated across a range of feature extraction tasks, including image classification, image reconstruction, and automatic feature learning. Comparative benchmarks against conventional neural networks demonstrate that our approach achieves comparable performance while offering significant implementation advantages. In addition, we systematically assess the robustness of the method under a variety of noisy conditions and device-level imperfections, confirming its resilience and adaptability. These qualities make projection-based activation particularly well suited for practical deployment in deep ONNs, with promising applications in areas such as image sensing and real-time optical information processing^29–31^.

## Results and discussions

### Complex-valued neural network (CVNN) with projection layers

**Fig. 1:**
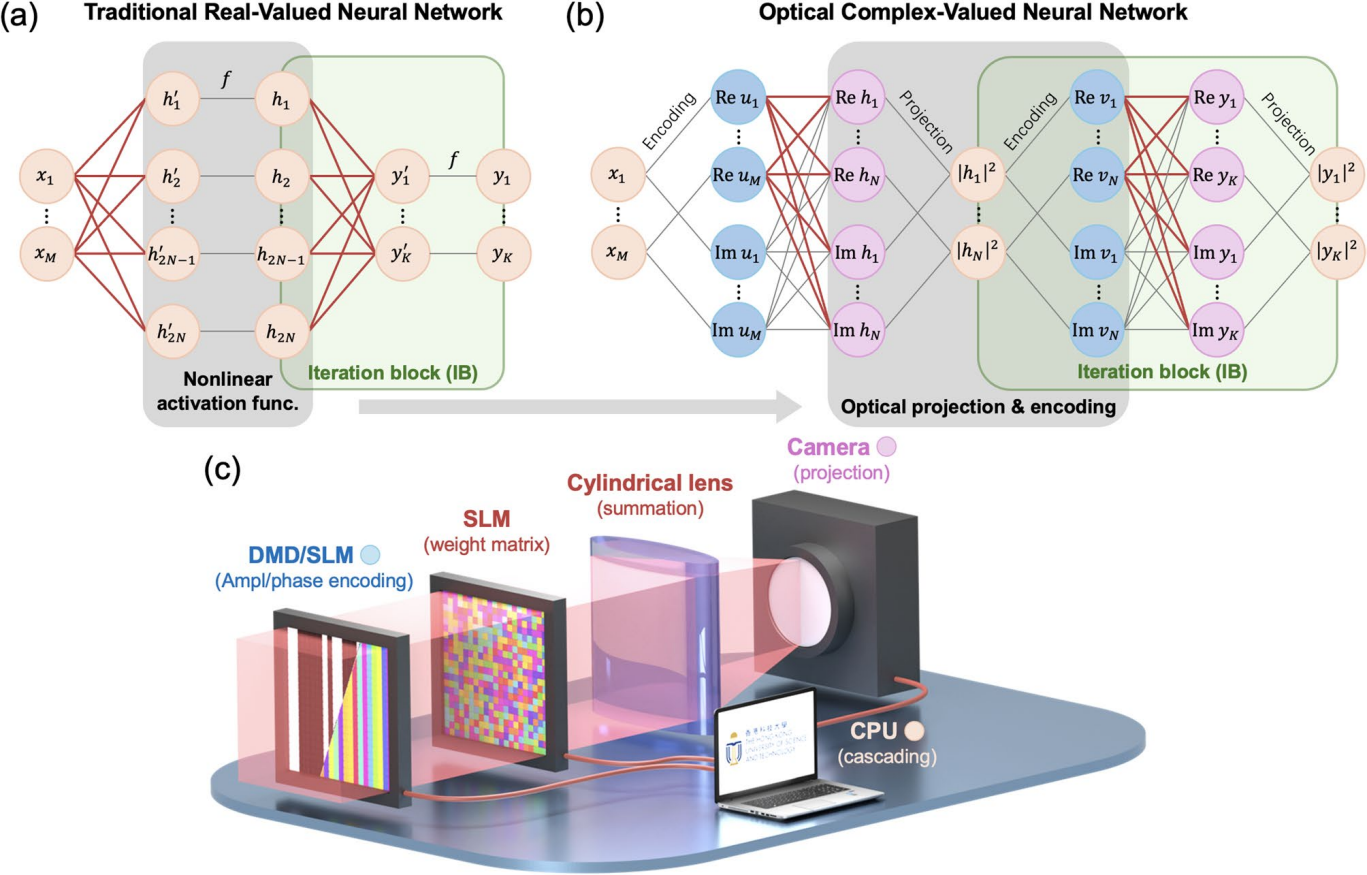
Schematic of the complex-valued neural network (CVNN) with projection layers and the experimental implementation setup. (**a**) Schematic of a traditional RVNN utilizing predefined nonlinear activations, consisting of two iteration blocks (IBs). (**b**) Schematic of the CVNN with projection layers, also comprising two IBs. The input can be encoded as either amplitude or phase profile of the electric field. By applying the complex weight matrix, the complex-valued output is generated and subsequently projected onto the intensity. This projection functions, together with encoding, as a nonlinear activation mechanism analogous to that in traditional RVNNs. (**c**) Experimental setup for implementing the optical CVNN with projection layers. The input is generated using a digital micromirror device (DMD) or spatial light modulator (SLM) for amplitude or phase encoding, respectively. The second SLM implements the complex weight matrix. The intensity of the electric field is measured by a camera, which can be used as the input for the subsequent IB through cascading.

We begin by outlining the operational framework of the proposed ONNs incorporating projection layers. For clarity and comparison, we first introduce a conventional real-valued neural network (RVNN) with a single hidden layer, as illustrated in Fig. 1(a). This RVNN comprises fully connected layers (FCLs), with predefined activation functions applied after the hidden and output layers to introduce nonlinearity. The input is a real-valued vector ${X}_{M}=\{{x}_{1},{x}_{2},\dots ,{x}_{M}\}$, which is transformed by the hidden layer and the first activation function $f$ (depicted as the gray box in Fig. 1(a)) into a real-valued output vector ${H}_{2N}=\{{h}_{1},{h}_{2},\dots ,{h}_{2N}\}$, where $M$ and $2N$ denote the lengths of the input and hidden layer output vectors, respectively. This transformation can be expressed as ${H}_{2N}=f({W}_{2N\times M}{X}_{M}+{B}_{2N})$, where ${W}_{2N\times M}$ and ${B}_{2N}$ are the trainable real-valued weight matrix and bias vector (indicated by red lines in Fig. 1(a)). We refer to this mapping from ${X}_{M}\to {H}_{2N}$ as an iteration block (IB), which can be cascaded to construct deeper RVNNs. For example, as shown in Fig. 1(a), the output ${H}_{2N}$ can serve as the input to a second IB, yielding a final real-valued output vector ${Y}_{K}=\left\{{y}_{1},{y}_{2},\dots ,{y}_{K}\right\}$, resulting in a two-layer RVNN.

We now present an equivalent implementation of the RVNN using a complex-valued neural network (CVNN) architecture composed of two iteration blocks, as illustrated in Fig. 1(b). In this CVNN framework, the initial input ${X}_{M}$ remains the same as in the RVNN. However, the network operates in the optical domain by representing data as complex-valued electric fields. To facilitate this, the input is first encoded into a complex field profile via a designated encoding function $\mathcal{E}$, such as amplitude or phase encoding (with later description), yielding ${U}_{M}=\left\{{u}_{1},{u}_{2},\dots ,{u}_{M}\right\}=\mathcal{E}\left({X}_{M}\right)$. Subsequently, a spatial modulation is applied to the encoded field profile to produce the transformed complex field ${H}_{N}=\left\{{h}_{1},{h}_{2},\dots ,{h}_{N}\right\}$, via a complex-valued matrix–vector multiplication: ${H}_{N}={W}_{N\times M} {U}_{M}$, where ${W}_{N\times M}$ is a trainable complex-valued weight matrix. In our numerical implementation, the CVNN consists of fully connected layers without bias terms. To enable compatibility with real-valued computation frameworks and optical implementations, all complex variables are decomposed into their real and imaginary components, as shown in Fig. 1(b). The complex matrix–vector multiplication can then be expressed in real-valued block matrix form as:

1 $$\left(\begin{array}{c}\text{Re} {H}_{N}\\ \text{Im} {H}_{N}\end{array}\right)=\left(\begin{array}{cc}\text{Re} {W}_{N\times M}& -\text{Im} {W}_{N\times M}\\ \text{Im} {W}_{N\times M}& \text{Re} {W}_{N\times M}\end{array}\right) \left(\begin{array}{c}\text{Re} {U}_{M}\\ \text{Im} {U}_{M}\end{array}\right).$$

This fully connected layer (FCL) has input and output dimensions of $2M$ and $2N$, respectively, but requires only $2NM$ trainable parameters (indicated by the red lines in Fig. 1(b)). This parameter efficiency is achieved by enforcing the symmetry structure described in Eq. (1), which arises naturally from the real-imaginary decomposition of the complex weight matrix.

After a layer of complex matrix–vector multiplication, we assume the complex-valued output ${H}_{N}$ is captured by an optical detector such as a camera, which performs a projection from the complex electric field to a real-valued intensity profile ${\left|{H}_{N}\right|}^{2}$. We refer to this intensity measurement stage as a projection layer. Analogous to the RVNN structure in Fig. 1(a), the complete sequence in Fig. 1(b)—comprising input encoding (${X}_{M}\to {U}_{M}$), field transformation (${U}_{M}\to {H}_{N}$), and projection (${H}_{N}\to {\left|{H}_{N}\right|}^{2}$)—constitutes a single iteration block (IB). The resulting intensity vector ${\left|{H}_{N}\right|}^{2}$ can then serve as the input to the next IB, yielding a final output vector ${\left|{Y}_{K}\right|}^{2}=\{{\left|{y}_{1}\right|}^{2},{\left|{y}_{2}\right|}^{2},\dots ,{\left|{y}_{K}\right|}^{2}\}$. Notably, both the input encoding and the projection processes introduce effective nonlinearity into the network, as indicated by the gray box in Fig. 1(b), playing a role analogous to the activation function in Fig. 1(a).

To evaluate the effectiveness of the nonlinearity introduced by our optical CVNN framework, we benchmark its performance against that of a conventional RVNN. Based on the input and output dimensions of each iteration block, the CVNN architecture shown in Fig. 1(b) can be characterized by the structure $M-N-K$. The total number of trainable parameters in this network corresponds to the two complex-valued weight matrices ${W}_{N\times M}$ and ${W}_{K\times N}$, each contributing real and imaginary components. As a result, the total number of trainable real-valued parameters in the CVNN is $2(N\times M)+2(K\times N)$. For a fair comparison, the RVNN in Fig. 1(a) is configured to have the same input and output dimensions as the CVNN. To match the parameter count, the width of the first hidden layer in the RVNN is set to $2N$, leading to a total of $\left(2N\times M+2N\right)+(K\times 2N+K)$ trainable parameters, including both weights and biases for the two fully connected layers. This setup ensures that the comparison reflects architectural differences—particularly the role of the projection as effective nonlinearity—rather than discrepancies in model capacity (number of weights and biases to train).

### Optical implementation of CVNN

Figure 1(c) shows a schematic representation of an optical implementation of CVNN. This setup employs a DMD or a SLM to encode the input vector ${X}_{M}$ as either an amplitude or phase profile, denoted as ${U}_{M}$. In the proposed scheme, the encoded field profile ${U}_{M}$ is spatially distributed as an irregular grating along the horizontal axis. This input field ${U}_{M}$, encoded by the DMD or SLM, is then modulated by a second SLM capable of both amplitude and phase modulation. This SLM implements the complex weight matrix ${W}_{N\times M}$ within a 4f. optical system. Specifically, each row of ${W}_{N\times M}$ is spatially laid out along the horizontal direction of the SLM, enabling elementwise multiplication in accordance with Eq. (1). Each input component ${u}_{l}$ is modulated into ${w}_{nl}{u}_{l}$, where *l* and $n$ index the input and output neurons, respectively. To perform the summation following this elementwise multiplication, a cylindrical lens is used. At its focal plane, the lens performs a one-dimensional Fourier transform along the horizontal axis, such that the central vertical line of the output field is proportional to the sum ${h}_{n}=\sum_{l=1}^{M}{w}_{nl}{u}_{l},$ as demonstrated in previous works^27,28^. This combination of spatial modulation and Fourier-domain summation effectively realizes the linear electric field transformation ${U}_{M}\to {H}_{N}$. Following this transformation, a camera captures the complex field ${H}_{N}$ and projects it into an intensity profile ${\left|{H}_{N}\right|}^{2}$, which serves as the real-valued output of the projection layer. The complete sequence of encoding, field transformation, and projection thus constitutes a single IB. For amplitude encoding, |H_N| is used as the real part of complex value input of next layer. For phase encoding, $\left|{H}_{N}\right|$ is used to set linearly the argument of the complex value input (with unit magnitude) of next layer. We note that the effective nonlinearity comes from both the projection and the encoding scheme (see Supplementary Information for more details). Subsequent IBs can be implemented using the same optical hardware, with scalability achieved through temporal multiplexing, allowing deeper ONNs to be constructed within a limited set of optical components^11,12^. This experimental setup leverages intensity measurement with encoding as a nonlinear activation mechanism, thereby eliminating the need for more complex or inefficient methods for achieving optical nonlinearity^10,22,26^. We note that instead of using a DMD with an SLM for amplitude and phase control of the input field, it is also possible to use two SLMs together^32^ or a single SLM with a macro-pixel approach by grouping pixels^33^. In all these cases, however, there is a compromise, either in the form of reduced resolution or in the requirement for precise alignment.

### CVNN for image classification and reconstruction

**Fig. 2:**
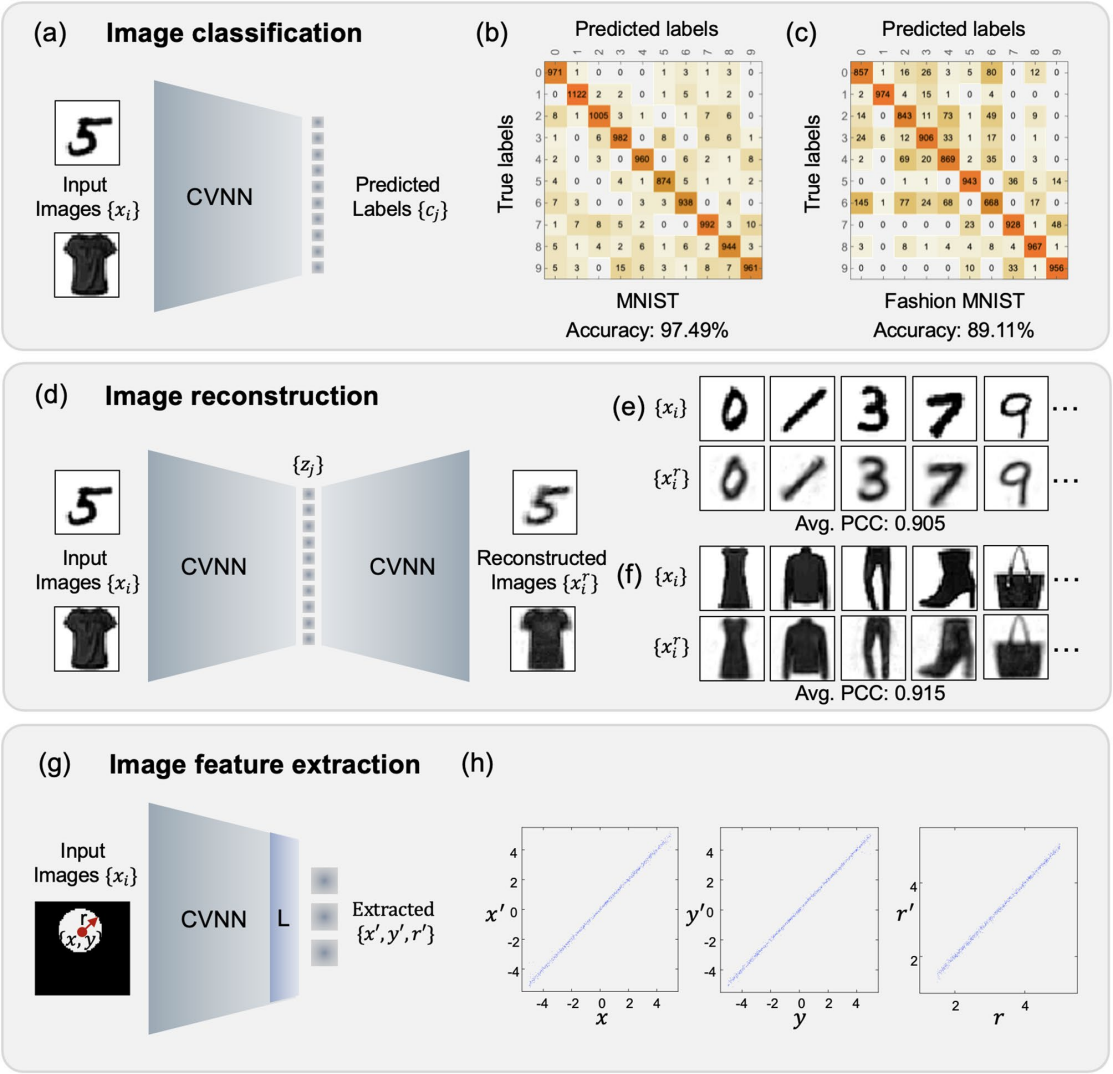
Implementations of CVNN with complex projection layers on feature extraction tasks across three different levels. (**a**) Schematic of the network for image classification. (**b**)-(**c**) Testing results for classification on (b) MNIST dataset and (**c**) Fashion MNIST dataset, with the averaged accuracy of 97.49% and 89.11%, respectively. (**d**) Schematic of the network for image reconstruction. (**e**)-(**f**) Testing results for image reconstruction on (e) MNIST dataset and (**f**) Fashion MNIST dataset. (**g**) Schematic of the network for the specific image feature extraction. The input image is a white circle in a square box. The radius *r* and position (*x, y*) of the circle can be varied. By incorporating a digital linear layer (denoted as “L”) into the CVNN, the trained network is capable of extracting the three features embedded in the input image. (**h**) Testing results for image feature extraction.

To validate the efficacy of the proposed complex-valued neural network (CVNN) integrated with projection layers, we evaluated its performance across three hierarchical feature extraction tasks: image classification, image reconstruction, and specific image feature extraction, as illustrated in Fig. 2. In all cases, grayscale images were encoded as amplitude profiles of light, serving as input to the network. For the image classification task, the network is designed to extract class-representative features from input images, with output categories encoded as discrete one-hot vectors. As shown in Fig. 2(a), the CVNN was trained independently on two benchmark datasets—MNIST and Fashion-MNIST—to assess its versatility and robustness across different data distributions. Each input image $\{{x}_{i}\}$ was resized to 32 × 32, flattened, and mapped to a class label represented by a one-hot vector $\{{c}_{j}\}$. The network architecture consisted of four IBs with a layer structure of 1024–64–64–64–10. Further details of the network design and training procedures are provided in Table S1. The performance of the trained CVNN was assessed using confusion matrices computed on the test sets of both datasets, with results shown in Figs. 2(b) and (c). The network achieved average classification accuracies of 97.49% on MNIST and 89.11% on Fashion-MNIST, demonstrating the network’s capability to discern and classify diverse datasets with notable precision.

In the image reconstruction task, the network is trained to map each input image $\{{x}_{i}\}$ to a set of ten latent variables $\{{z}_{j}\}$, which encode continuous-valued features for reconstructing the input, as illustrated in Fig. 2(d). In contrast to the classification task, where the outputs $\{{c}_{j}\}$ represent discrete category labels (Fig. 2(a)), the latent variables $\{{z}_{j}\}$ capture more subtle and continuous characteristics such as writing style, stroke thickness, and image clarity. Using the same training datasets—MNIST and Fashion-MNIST—the CVNN is constructed with four iteration blocks and a symmetric layer structure of 1024–64–10–64–1024. The network is trained under the same optimization settings as in the classification task, but now using an unsupervised learning approach. Figures 2(e) and (f) display representative testing examples and their reconstructions from the MNIST and Fashion-MNIST datasets, respectively. To quantify the reconstruction quality, we compute the average Pearson correlation coefficient (PCC) between the original images $\{{x}_{i}\}$ and the reconstructed outputs $\{{x}_{i}^{r}\}$, obtaining scores of 0.905 and 0.915 for MNIST and Fashion-MNIST, respectively. These results demonstrate the capability of the proposed CVNN to effectively encode and reconstruct input images with high fidelity.

### CVNN for supervised feature extraction

To further demonstrate the versatility of the proposed approach, we apply it to a specific image feature extraction task by introducing an additional digital output layer. As illustrated in Fig. 2(g), the network is trained to extract three continuous parameters: the coordinates $(x,y)$ and the radius $r$ of a circle inscribed within a square of 10 cm width. Similar to the reconstruction task in Fig. 2(d), the extracted features are continuous; however, this task is performed using supervised learning, since the ground truth feature values are known. Each input image is of dimension 1024 (flattened from 32 by 32) and represents a square containing a circle with randomly generated parameters. Specifically, the coordinates $x$ and $y$ are sampled from a uniform distribution U[− 5,5] cm, and the radius $r$ is sampled from U[1.5,5] cm. Then, a total of 10,000 images of 32-by-32 pixels are generated with a white circular disk of a specific radius and location within a black background, with 81% used for training, 9% for validation, and the remaining 10% of these images for testing. This corresponds to an average nearest neighbor distance, interpreted as a resolution, for the training data around 0.194 cm, according to the Poisson point process. The network architecture consists of four iteration blocks followed by a digital output layer (denoted as “L” in Fig. 2(g)), yielding a layer structure of 1024–64–64–64–64–3. Additional design and training details are provided in Table S1. As shown in Fig. 2(h), the predicted parameters $\{{x}{\prime},{y}{\prime},{r}{\prime}\}$ align closely with the ground truth, achieving an average PCC of 0.999. This result highlights the model’s ability to perform high-precision regression tasks using the proposed CVNN framework. Notably, to show the trained network has the ability of generalizing, we construct an out-of-distribution test dataset with $x$=$y$=0 cm and $r$ sampled from U[^5, 10^] cm. The model achieves a PCC of 0.994 between predicted and ground-truth radius, demonstrating generalization.

To assess the noise robustness of the proposed optical CVNN in realistic settings, we introduce controlled imperfections into the trained networks from Fig. 2, simulating various noisy environments. In particular, we apply additive noise to the complex-valued weight matrices by perturbing each element ${w}_{nl}$ with a complex-valued bias ${\varepsilon }_{nl}$, such that ${w}_{nl}\to {w}_{nl}+{\varepsilon }_{nl}$, where the real and imaginary components of ${\varepsilon }_{nl}$ are independently drawn from a normal distribution $N(0,{\sigma }_{noise}^{2})$. In practical optical implementations, such additive noise may arise from sources such as ambient light interference or inaccuracies in device calibration^27^. Moreover, we evaluate the effects of network depth and the slope of activation function (projection layer) on noise robustness^34,35^. We conduct tests by injecting additive noise selectively into individual layers and uniformly across all layers for varying values of ${\sigma }_{noise}$, with the resulting performance summarized in Figures S2–S5. The results show that while performance naturally degrades with increasing noise levels, deeper networks tend to exhibit greater robustness. Moreover, the noise tolerance is influenced by the slope of the activation function, highlighting the interplay between projection characteristics and network stability. Further details and expanded discussion are provided in the Supplementary Information.

### Possible extension to quaternion-valued neural network for projection layer

We highlight that the proposed projection layer is not restricted to the two-dimensional complex domain, consisting of real and imaginary components as shown in Fig. 1(b). It can be naturally extended to the quaternion domain, a four-dimensional hypercomplex space. In this case, quaternion is a hyper complex number with four real numbers as the components of a quaternion, instead of two real numbers in the case of conventional complex numbers. In this case, being written in analogous to the complex-valued formulation in Eq. (1), a quaternion matrix–vector multiplication can be expressed using a block matrix representation as follows:

2 $$\left(\begin{array}{c}\begin{array}{c}{Y}_{N}^{r}\\ {Y}_{N}^{i}\end{array}\\ \begin{array}{c}{Y}_{N}^{j}\\ {Y}_{N}^{k}\end{array}\end{array}\right)=\left(\begin{array}{cc}\begin{array}{cc}{W}_{N\times M}^{r}& -{W}_{N\times M}^{i}\\ {W}_{N\times M}^{i}& {W}_{N\times M}^{r}\end{array}& \begin{array}{cc}-{W}_{N\times M}^{j}& -{W}_{N\times M}^{k}\\ -{W}_{N\times M}^{k}& {W}_{N\times M}^{j}\end{array}\\ \begin{array}{cc}{W}_{N\times M}^{j}& {W}_{N\times M}^{k}\\ {W}_{N\times M}^{k}& -{W}_{N\times M}^{j}\end{array}& \begin{array}{cc}{W}_{N\times M}^{r}& -{W}_{N\times M}^{i}\\ {W}_{N\times M}^{i}& {W}_{N\times M}^{r}\end{array}\end{array}\right) \left(\begin{array}{c}\begin{array}{c}{X}_{M}^{r}\\ {X}_{M}^{i}\end{array}\\ \begin{array}{c}{X}_{M}^{j}\\ {X}_{M}^{k}\end{array}\end{array}\right).$$

Here, the superscript $r$ denotes the real part of the quaternion, while $i$, $j$, and $k$ correspond to its three imaginary components. The projection layer performs an effective nonlinear operation by computing the squared amplitudes of these components and summing them. Specifically, the quaternion projection is defined as $\left\{{Y}_{N}^{r},{Y}_{N}^{i},{Y}_{N}^{j},{Y}_{N}^{k}\right\}\to |{Y}_{N}^{r}{\left.\right|}^{2}+ |{Y}_{N}^{i}{\left.\right|}^{2}+|{Y}_{N}^{j}{\left.\right|}^{2}+|{Y}_{N}^{k}{\left.\right|}^{2}$. This summation yields a real-valued intensity profile, generalizing the projection mechanism from complex to quaternion-valued domains (see Fig. S1 for more details). Notably, quaternion-based approaches in optical signal processing allow the simultaneous encoding of amplitude, phase, and polarization of light within a unified quaternion-valued representation^36^, enabling more accurate modeling of polarization characteristics in optical signals. In contrast to conventional real-valued convolutional neural networks, which often compress multi-channel inputs and overlook inter-channel dependencies, quaternion convolutional neural networks preserve these relationships by treating inputs as structured quaternion matrices^37^. This formulation not only enhances the network’s expressiveness in capturing polarization dynamics but also offers computational efficiencies by reducing parameter redundancy and improving training convergence^38^.

### Benchmarking CVNNs with projection layers against conventional RVNNs

**Fig. 3:**
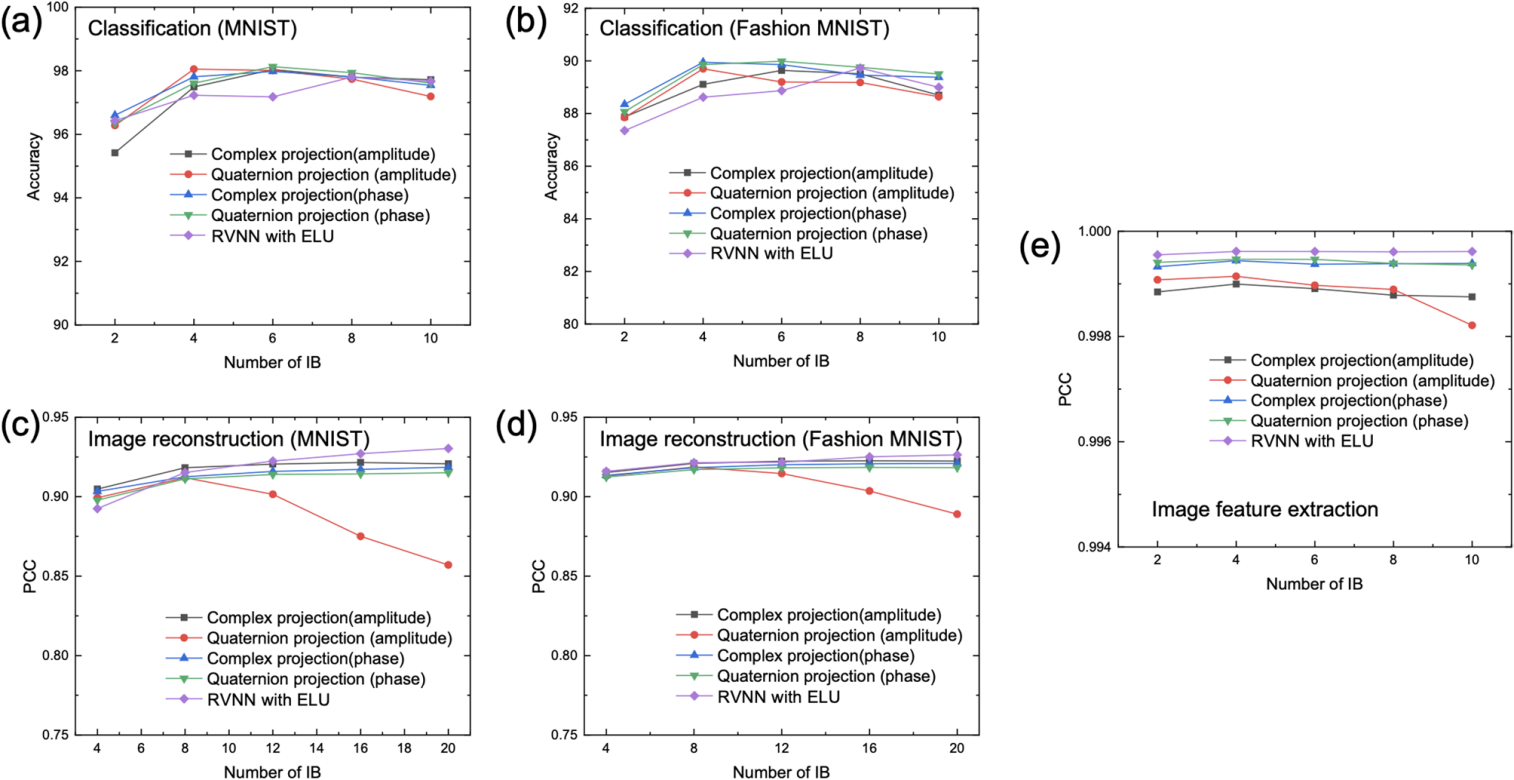
Performance comparison between the CVNN and traditional RVNN using different numbers of IBs across different applications. (**a**)-(**b**) Testing accuracies of different approaches in classification tasks on (**a**) MNIST dataset and (**b**) Fashion MNIST dataset. (**c**)-(**d**) Testing PCCs of different approaches in image reconstruction tasks on (**c**) MNIST dataset and (**d**) Fashion MNIST dataset. (**e**) Testing PCCs for image feature extraction tasks using different approaches.

Having validated the nonlinearity and effectiveness of the projection layer, we now compare the performance of the proposed CVNN with that of a traditional RVNN across the three previously described tasks. For each task, we employ the same training datasets as those used in Fig. 2, but adapt the network architectures with varying numbers of iteration blocks and structural configurations appropriate for complex and quaternion-valued networks. To ensure a fair comparison, both the CVNN and RVNN are designed to have a comparable number of trainable parameters, as detailed in Table S2. For clarity, we note that all RVNN and CVNN results reported in this study are obtained from numerical simulations. In the CVNN models, inputs are numerically encoded as either amplitude or phase profiles and processed using either complex or quaternion projections. For each task, both RVNN and CVNN are trained with identical datasets and hyperparameters and evaluated numerically on the same testing data. The corresponding testing results are summarized in Fig. 3. Across all tasks, the CVNN with projection layers achieves classification accuracies and PCCs comparable with, and in some cases superior to, those of the RVNN using an exponential linear unit (ELU) as its nonlinear activation function. Notably, in the image classification task, the CVNN with both complex and quaternion projections outperforms the RVNN in testing accuracy.

### CVNN for unsupervised feature extraction using variational autoencoder

To further demonstrate the effectiveness and scalability of the projection layer, we extend its application to a more advanced level of feature extraction by integrating it into an unsupervised learning framework based on the β-Variational Autoencoder (β-VAE). To maintain generality and highlight the broader applicability of the method, we apply the β-VAE architecture to a scalar-wave propagation model in a lossy medium, aiming to infer system parameters directly from the observed wave responses. As a representative example, we adopt a spring–mass lattice model, which is sufficiently simple to allow clear interpretation and has been extensively studied in a previous work using traditional RVNNs^39^. This model serves as a benchmark to evaluate the capability of the CVNN with projection layers in capturing latent physical parameters in a completely unsupervised manner. The incorporation of the projection mechanism within the β-VAE demonstrates its suitability for parameter inference tasks in complex physical systems, further supporting its potential in broader applications beyond standard image-based datasets.

**Fig. 4:**
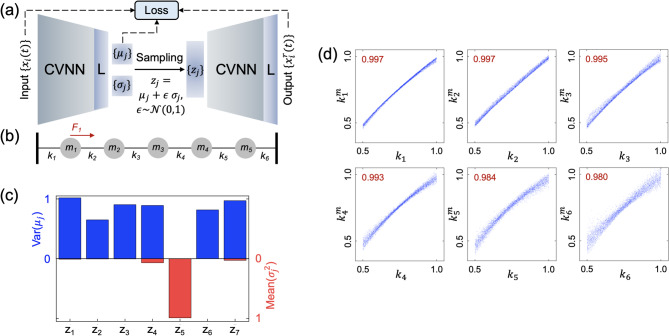
The implementation of CVNN with projection layers on $\beta$-VAE. (**a**) The architecture of $\beta$-VAE using CVNN with projection layers and digital layers. (**b**) The schematic of the spring-mass system. (**c**) The performance of the extracted latent variables with 6 meaningful variables. (**d**) Extracted spring constants ${k}_{i}^{m}$ versus true spring constants ${k}_{i}$ for the testing data. The corresponding PCCs are labeled in red.

As shown in Fig. 4(b), the model consists of six springs with spring constants ${k}_{i}$ and five oscillators with masses ${m}_{i}$, arranged in a one-dimensional chain and connected to their nearest neighbors. Boundary springs are attached to rigid walls. A unit impulse excitation ${F}_{1}$ is applied to the first oscillator, and the resulting displacements $X=\{{x}_{i}(t)\}$ are obtained by numerically solving the governing equation of motion:

3 $${\varvec{M}}{\partial }_{t}^{2}X+b{\partial }_{t}X+{\varvec{K}}X={\varvec{F}},$$

where $b$ = 0.5 kg/s is the damping factor, identical for all oscillators. Here, ${\varvec{M}}$, ${\varvec{K}}$, and ***F*** denote the mass matrix, stiffness (spring constant) matrix, and external force vector, respectively, with their full expressions given in Eq. S1 of the Supplementary Information. In this task, the mass of each oscillator is fixed at 1 kg, while the spring constants $\{{k}_{i}\}$ are treated as the system parameters to be inferred or imaged from the observed displacement responses $X$. To generate the dataset, each spring constant is sampled independently from a uniform distribution U[0.5,1.0] $\text{kg}/{\text{s}}^{2}$, with mass fixed at 1 kg and displacement data for the first 10 s from an impulse excitation at the first node are generated from Eq. (3), resulting in a total of 50,000 simulated displacement profiles. Of these, 81% are used for training, 9% for validation, and the remaining 10% for testing. The displacements $\{{x}_{i}(t)\}$ are multivariate time-series inputs, with each of the nodes providing a 101-dimensional sequence (time step = 0.1 s over 10 s) and a total length of 505 data points for the time sequence of each sample.

As shown in Fig. 4(a), the β-VAE architecture consists of an encoder and a decoder, each comprising four IBs followed by a digital output layer. The encoder is designed to extract a low-dimensional, compressed representation of the input displacement data $\{{x}_{i}(t)\}$, storing the features in a latent space represented by variables $\{{z}_{j}\}$, with each latent variable ${z}_{j}$ assumed to satisfy distribution generated by ${\mu }_{j}+\epsilon {\sigma }_{j}$ where $\epsilon$ is the standard normal distribution. The decoder then reconstructs the displacement profile $\{{x}_{i}^{r}(t)\}$ from the sampled latent variables, aiming to approximate the original input data as closely as possible while the loss function of $\beta$-VAE is given by:

4 $${\left|\left|x-{x}^{r}\right|\right|}_{2}^{2}+\beta \sum_{n}{D}_{KL}[\mathcal{N}({\mu }_{n},{\sigma }_{n}^{2})||\mathcal{N}({\text{0,1}}^{2})].$$

The first term is the mean squared error (MSE) between the input and reconstructed displacements, and the second term is the Kullback–Leibler (KL) divergence, which enforces each latent variable ${z}_{j}$ to follow preferably a standard normal distribution $\mathcal{N}({\text{0,1}}^{2})$ in addition to minimizing reconstruction error. Additional details about the network structure and training parameters are provided in Table S1. Notably, the β-VAE framework is capable of learning a compact and interpretable representation of the displacement data. It also provides a means to evaluate the uniqueness and sufficiency of the inverse mapping from displacement responses to physical parameters—in this case, the spring constants. This makes it a valuable tool for assessing the feasibility of inverse imaging in complex physical systems.

Figure 4(c) presents the statistical parameters of the latent variables obtained after training the β-VAE. The latent dimension was manually set to seven. Among the latent variables, it is observed that ${z}_{5}$ carries no meaningful information, as evidenced by its negligible variance in ${\mu }_{5}$ and an average ${\sigma }_{5}$ close to one across the entire dataset, i.e. being random without definite value inferred from the displacement data^40^. In contrast, the remaining six latent variables encode essential information, corresponding precisely to the six degrees of freedom to be imaged in the system—i.e., the six independently varied spring constants in the spring–mass model. Each of these informative latent variables can be interpreted as a linear combination of the six spring constants, consistent with findings reported in previous studies^37^. When the trained network is applied to test data, the resulting latent variables are linearly transformed into predicted spring constants $\{{k}_{i}^{m}\}$, as shown in Fig. 4(d). The PCCs between the predicted values $\{{k}_{i}^{m}\}$ and the ground truth $\{{k}_{i}\}$ all exceed 0.980, confirming the model’s ability to accurately infer the physical parameters from input displacement data. Compared with a β-VAE implemented using RVNN and trained on the same datasets, CVNN achieves comparable average PCCs across the six spring constants (CVNN:0.991, RVNN: 0.995), confirming that it provides faithful performance for β-VAE implementation. To examine the effect of lower data resolution, we train the CVNN on only 18% of the samples (9,000). The mean PCC across the six spring constants decreases slightly from 0.991 to 0.974, while all values remain above 0.905, indicating that the model captures certain interpolation and generalization capability out of the training data set.

Notably, both the encoder and decoder in Fig. 4(a) consist of four IBs followed by a digital output layer (“L”). The key difference is that IBs contain no bias weights and include a projection layer, whereas the digital layer is required to produce the final outputs ${\mu }_{j}$ and $\text{log}{\sigma }_{j}$ in the β-VAE. While the optical implementation substantially reduces the computational cost of the IBs, the digital layer remains necessary for stochastic operations such as random sampling in the latent space in this application. To evaluate how closely the digital layer can be aligned with IB functionality, we retrained the network with bias weights disabled in the digital layer “L”. The performance remained nearly unchanged, with only a slight drop in average PCC (from 0.991 to 0.989 across the six spring constants). This result suggests that CVNN can be pushed further toward a fully optical implementation without appreciable performance degradation.

## Conclusions

In conclusion, we have systematically investigated the use of projection layers, together with amplitude or phase encoding, as alternatives to conventional nonlinear activation functions for constructing ONNs, extending the concept to include quaternion projections. Through numerical experiments and comparative evaluations, we have validated the effectiveness and robustness of this approach across a range of feature extraction tasks, including image classification, image reconstruction, and physical parameter inference. The proposed method achieves performance comparable to traditional networks with standard activation functions, while offering advantages in scalability and physical realizability. Unlike previous intensity-based ONNs, which employed photodetection only at the final output layer, our framework promotes the projection operation itself to a differentiable, layer-wise activation compatible with back-propagation and scalable optical implementation. In addition to leveraging the benefits of optical computing, this work highlights the potential of exploiting the multi-component nature of quaternions and projection layers as a foundation for non-linear operations in deep learning models. The integration of optical signal processing with deep neural networks presents a promising framework for next-generation computing systems, enabling improvements in energy efficiency, speed, and representational capacity. This approach opens the door to more efficient and powerful optical-based learning systems with wide-ranging applications in image recognition, signal processing, and beyond^19,41,42^, and provides a systematic benchmark for projection-based nonlinearities in every intermediate layer. By exploring both amplitude and phase encoding pathways—necessary for cascading multiple layers—we demonstrate that projection-based nonlinearities can be effectively used as activations without significantly degrading network performance. This establishes a pathway toward deeper optical network architectures and highlights future opportunities for optoelectronic adaptation that leverage the inherent parallelism of photonic systems. Furthermore, compared with material-based optical nonlinearities (e.g., the Kerr effect^18,19,21^), which offer fast response but require resonant enhancement, thermal stabilization, and high optical intensity, our projection-based approach achieves nonlinearity through linear optics and detection. This preserves the low-power advantage of optical computing and complements diffractive systems such as D^2^NNs in extending network complexity.

It is worth mentioning that while optical implementation offers huge parallelism for data processing, our approach—although repeatedly using the same setup offers scalability to multiple layers—also introduces latency during the conversion between analog and digital signals. Our approach is currently limited by modulator refresh rates—about 1 kHz for advanced SLMs and up to 20 kHz for high-speed DMDs^27^—corresponding to sub-millisecond latency per layer. Digital data-acquisition and processing electronics (such as FPGAs) already operate at MHz sampling rates, and high-speed cameras reach around 100 kHz, so the bottleneck mainly lies on the modulator side. This latency is expected to decrease as optoelectronic interfaces (e.g., high-speed cameras, modulators, and ADC/DACs) continue to improve, narrowing the gap observed in other temporally multiplexed optical-computing frameworks.

## Supplementary Information

Below is the link to the electronic supplementary material.

Supplementary Material 1

## Data Availability

The datasets used and/or analyzed during the current study are available from the corresponding author on reasonable request.
