# Supplementary material for: Optical neural networks with intensity‑based projection layers as effective nonlinear activations
Source: Sci Rep. 2025 Dec 9;16:1068. doi: 10.1038/s41598-025-30632-y (PMC12783648; doi:10.1038/s41598-025-30632-y)
Supplement: Supplementary file 1 — Supplementary Material 1 [file 41598_2025_30632_MOESM1_ESM.pdf]

Supplementary Information for

Optical Neural Networks with Intensity-Based Projection Layers as Effective Nonlinear Activations

1. Network structures of the complex-valued network (CVNN) with complex projection layers

The network architectures and training configurations utilized in Figures 2 and 4 across various applications are detailed in Table S1. In these implementations, the input to each iteration block (IB) is encoded as the amplitude profile of light. Notably, in the case of the CVNN, the input can be encoded based on either the amplitude or phase of light. In our implementation, amplitude encoding represents each input element as a real number normalized within the range of 0 to 1. For phase encoding, each input element is normalized within the range of 0 to π, denoted as θ, with its cosine and sine values representing the real and imaginary components of a complex number $e^{i\theta}$, respectively.

For the image classification (Figure 2(a)) and reconstruction (Figure 2(d)) tasks, the networks are composed of four IBs, with the amplitude of the output from the final IB serving as the final output. Besides, the networks for image feature extraction (Figure 2(g)) and automatic feature extraction using β-VAE (Figure 4(a)) consist of a series of IBs, with a digital layer serving as the output. Additionally, given the constraints imposed by optical devices, such as spatial light modulators (SLMs), on the implementation of the trainable weight matrix, we limit the amplitude of each complex-valued matrix element to less than 1 during training. The loss function employed for training the networks in Figure 2 is the mean-squared error, while the β-VAE in Figure 4 is trained using the loss function described in Equation 3 of the main text. All the networks were trained by using a computer with a single GPU (RTX 3090, 24GB).

Task	Network	Learning rate	Optimizer	Batch size	Epochs	β
Image Classification	1024-64-64-64-10	0.0001	Adam	500	500	-
Image reconstruction	1024-64-10-64-1024	0.0001	Adam	500	2500	-
Image Feature Extraction	1024-64-64-64-64-3	0.0001	Adam	500	2000	-

**Automatic Feature Extraction (β-VAE)**	Encoder: 505-50-50-50-50-7 Decoder: 7-50-50-50-50-505	0.0002	Adam	1000	5000	0.00015

Table S1. The network architectures and training configurations utilized in Figures 2 and 4 across various applications.

2. Schematic of CVNN with quaternion projections

We extend the concept of complex projection presented in Figure 1(b) to quaternion projection. As illustrated in Figure S1, the initial input X_M is first encoded using a designated encoding function (e.g. amplitude/phase encoding) $\mathcal{E}$: $\mathcal{E}(X_M) = U_M = \{u_1, u_2, \ldots, u_M\}$, mapping the input X_M to a set of quaternion numbers U_M. Subsequently, a quaternion matrix-vector multiplication is performed, represented as $H_{N/2} = W_{\frac{N}{2}\times M}\, U_M = \{h_1, h_2, \ldots, h_{N/2}\}$. The real-valued intensity profile $|H_{N/2}|^2$ is then calculated, serving as a quaternion projection layer. Subsequently, the resulting $|H_{N/2}|^2$ can be utilized as the input to the next IB to obtain K real variables $|Y_K|^2$. In our implementation, the CVNN with quaternion projection layers is constructed with full connected layers, and the output is divided into four equal parts, each corresponding to one of the four components of the output quaternions. The process of quaternion matrix-vector multiplication is detailed in Equation 2 of the main text. Besides, to maintain the same input and output dimensions as the CVNN employing complex projections in Figure 1(b) while preserving the same number of trainable parameters, the output width of the first IB in Figure S1 is set to N/2. Consequently, the total number of trainable parameters is $2NM + 2KN$.

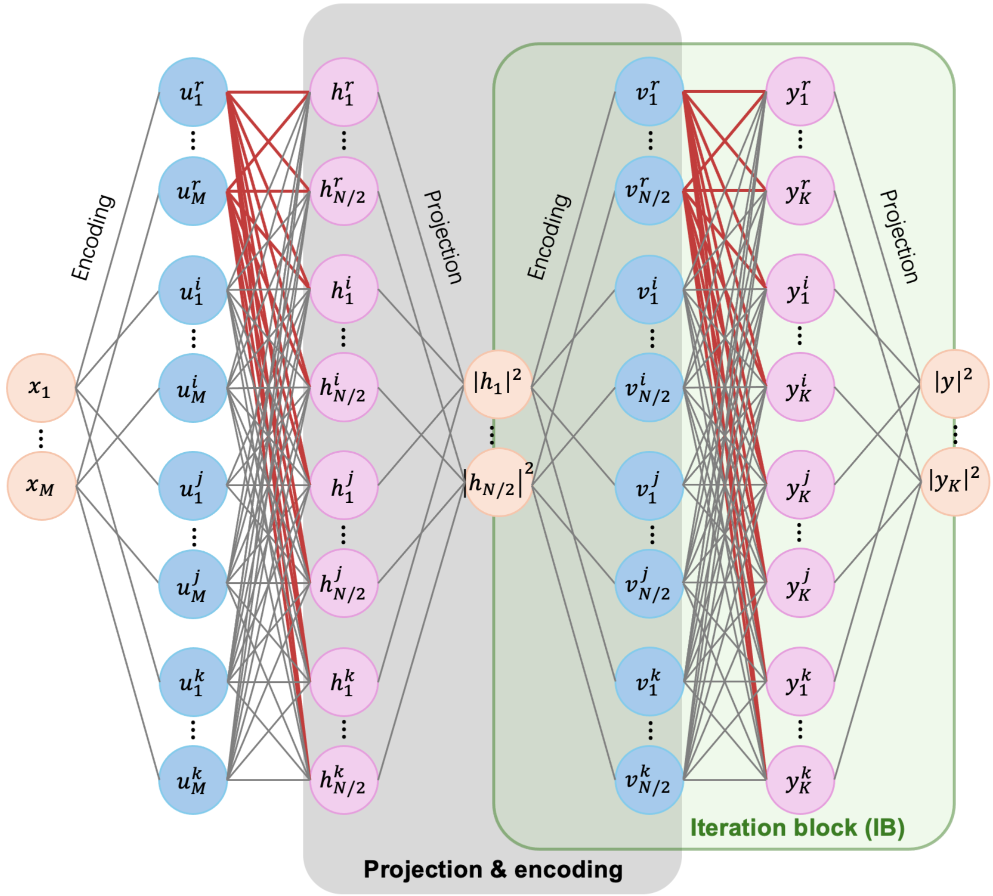

Figure S1. Schematic of CVNN with quaternion projections.

3. Network structure comparison of CVNN and RVNN

We compare the performance of the proposed CVNN method with that of traditional RVNN across three different tasks, as shown in Figure 3 in the main text. The network architectures with various IBs for these tasks are detailed in Table S2. Here, we use the CVNN employing complex projection layers as a benchmark. As discussed in Figure 1, to maintain consistent input and output dimensions while ensuring a comparable number of trainable parameters, we adjust the width of the IBs in both the traditional RVNN and the CVNN using quaternion projection layers. For example, in the image classification task, the input and output dimensions are fixed at 1024 and 10, respectively, and we construct the networks using an even number of IBs to ensure a comparable number of trainable parameters across different network configurations. For the image reconstruction task, the encoder structure mirrors that used for image classification, while the decoder has a reverse structure, resulting in the number of IBs being double that of the image classification networks. In the image feature extraction task, the number of IBs is even, followed by a digital layer to produce the output.

In the CVNN with quaternion projection layers, amplitude encoding represents each input element as a real number (utilizing the real part of the quaternion) normalized within the range of 0 to 1. Conversely, in phase encoding, each input element θ is normalized within the range of 0 to π, with its cosine and sine values representing the real and imaginary components of a quaternion number, respectively. Here, we utilize the real part and one imaginary component of the quaternion for simplicity (the encoded input is $\cos\theta + i\ \sin\theta$) in the encoding process, while all the quaternion weight matrix elements in the networks for both amplitude and phase encoding include all four components, which are optimized during training.

Task	Number of iteration blocks	CVNN with complex projection	CVNN with quaternion projection	RVNN with ELU activation function
**Image Classification**	2	1024-64-10	1024-32-10	1024-128-10
	4	1024-64-64-64-10	1024-32-64-32-10	1024-128-64-128-10
	6	1024-64-64-64-64-64-10	1024-32-64-32-64-32-10	1024-128-64-128-64-128-10
	8	1024-64-64-64-64-64-64-64-10	1024-32-64-32-64-32-64-32-10	1024-128-64-128-64-128-64-128-10
	10	1024-64-64-64-64-64-64-64-64-64-10	1024-32-64-32-64-32-64-32-64-32-10	1024-128-64-128-64-128-64-128-64-128-10
**Image reconstruction**	4	1024-64-10-64-1024	1024-32-10-32-1024	1024-128-10-128-1024
	8	1024-64-64-64-10-64-64-64-64-1024	1024-32-64-32-10-32-64-32-1024	1024-128-64-128-10-128-64-128-1024
	12	1024-64-64-64-64-64-10-64-64-64-64-64-1024	1024-32-64-32-64-32-10-32-64-32-64-32-1024	1024-128-64-128-64-128-10-128-64-128-64-128-1024
	16	1024-64-64-64-64-64-64-64-10-64-64-	1024-32-64-32-64-32-64-32-10-32-64-	1024-128-64-128-64-128-64-128-10-

		64-64-64-64-64-1024	32-64-32-64-32-1024	128-64-128-64-128-64-128-1024
	20	1024-64-64-64-64-64-64-64-64-64-10-64-64-64-64-64-64-64-64-64-1024	1024-32-64-32-64-32-64-32-64-32-10-32-64-32-64-32-64-32-64-32-1024	1024-128-64-128-64-128-64-128-64-128-10-128-64-128-64-128-64-128-64-128-1024
**Image Feature Extraction**	2	1024-64-64-3	1024-32-64-3	1024-128-64-3
	4	1024-64-64-64-64-3	1024-32-64-32-64-3	1024-128-64-128-64-3
	6	1024-64-64-64-64-64-64-3	1024-32-64-32-64-32-64-3	1024-128-64-128-64-128-64-3
	8	1024-64-64-64-64-64-64-64-64-3	1024-32-64-32-64-32-64-32-64-3	1024-128-64-128-64-128-64-128-64-3
	10	1024-64-64-64-64-64-64-64-64-64-64-3	1024-32-64-32-64-32-64-32-64-32-64-3	1024-128-64-128-64-128-64-128-64-128-64-3

Table S2. Network structures utilized in Figure 3 for performance comparison of the CVNN and traditional RVNN on different tasks.

4. Noise robustness testing of the proposed optical CVNN

To evaluate the noise robustness of the proposed optical CVNN for practical applications, we conduct numerical simulations by introducing additive noise into pre-trained CVNNs used for image classification (Figure 2(a) in the main text). In real-world conditions, such noise may result from ambient light or device calibration inaccuracies [1]. Here, additive noise is modeled by adding a complex-valued bias ε_{nl}, to each element of the complex-valued weight matrix w_{nl}, such that $w_{nl} \rightarrow w_{nl} + \varepsilon_{nl}$. The real and imaginary components of this noise are generated from a Gaussian distribution with zero mean and a standard deviation σ_{noise}. We define the noise power level as $\frac{\sigma_{noise}}{\sigma_w} \times 100\%$, where σ_w represents the weight standard deviation in the IB to which the noise is applied. We evaluate five noise power levels—1%, 5%, 10%, 15%, and 20%—with each level tested 20 times using different

random seeds, taking the average testing accuracy as the final metric. In addition, we evaluate the effect of additive noise on the pre-trained CVNNs with varying numbers of IBs (depth of the CVNN). For CVNNs trained for image classification, noise is introduced into the weights of all IBs. The results, shown in Figure S2(a), demonstrate a clear reduction in accuracy with increasing noise power level, with accuracy degradation generally more restricted in CVNNs of greater depth. To further benchmark network performance under noise, we employ two additional approaches for introducing noise [2]. First, additive noise is applied independently to the weights within each IB in the trained CVNN, and the normalized accuracy, defined as $\frac{1}{M}\sum_{i=1}^{M} A_i$, is shown in Figure S2(b), where A_i represents the testing accuracy with noise introduced in the i-th IB. Second, we introduce noise sequentially to the weights of the first N IBs, with the results shown in Figure S2(c). These analyses yield results consistent with those in Figure S2(a), further supporting the observed trend of accuracy degradation under increasing noise levels. Moreover, accuracy degradation is generally more constrained in CVNNs with greater depth [2].

In addition to network depth, we further investigate the impact of the activation function on noise robustness. Specifically, we define the activation function as $f(\mathrm{Re}\, H_N + i\, \mathrm{Im}\, H_N) = \alpha((\mathrm{Re}\, H_N)^2 + (\mathrm{Im}\, H_N)^2)$, where H_N denotes the complex electric field after the field transformation shown in Figure 1(b), and the slope $\alpha = 1$ corresponds to the proposed complex-valued projection layer. By varying the slope α, we aim to explore its effect, which can be associated with the gain or loss characteristics of the camera for intensity measurement. For all tests, we use pre-trained CVNNs with four IBs. Testing accuracies for different values of α under three noise application scenarios (the same as Figure S2)—noise added across all IBs, noise applied to individual IBs, and noise in the first N IBs—are presented in Figure S3. The findings indicate that accuracy degradation is indeed influenced by the activation function, with smaller values of α associated with increased noise robustness [3]. Furthermore, we apply the same noise robustness testing methods (Figures S2 and S3) to CVNNs trained for image reconstruction. The corresponding results, presented in Figures S4 and S5, reveal trends similar to those observed in the image classification task.

In the physical design of the optical system, the sensitivity to additive noise underscores the importance of precise calibration and minimizing ambient light exposure during practical experiments. Based on the results in Figures S2–S5, to enhance noise robustness, a deeper CVNN model can be

employed alongside a projection layer with a lower gain when mapping from complex-valued electric field inputs to intensity outputs.

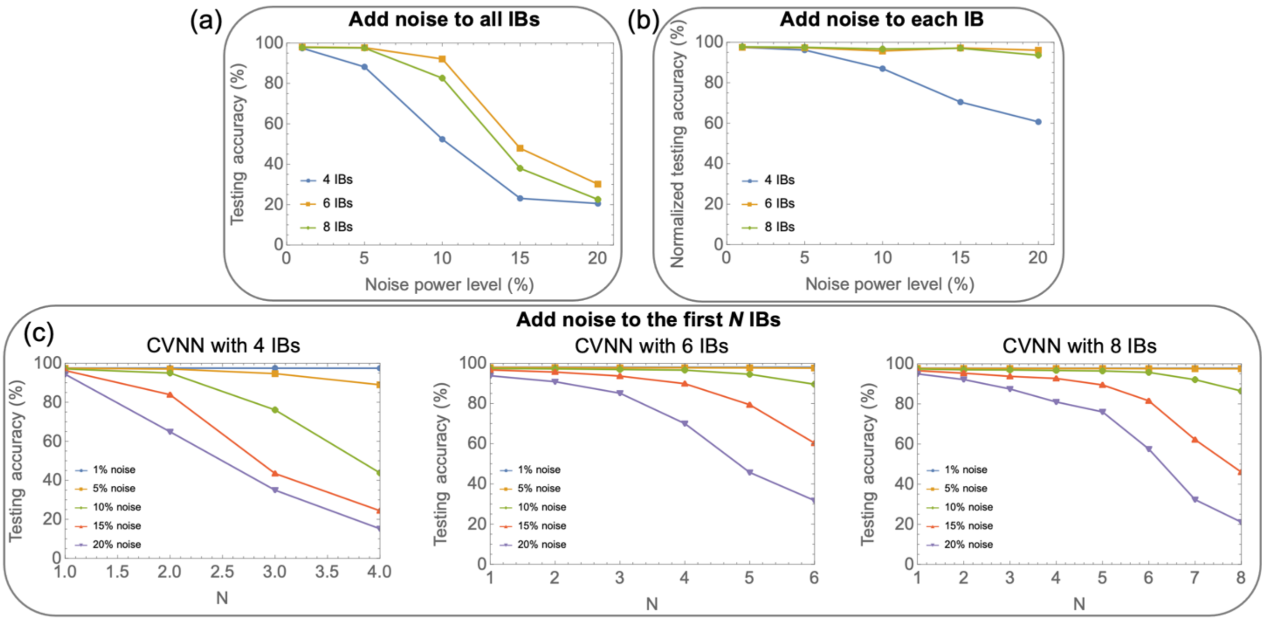

Figure S2. Testing performance of the trained CVNNs constructed with different number of IBs for image classification with different noise levels.

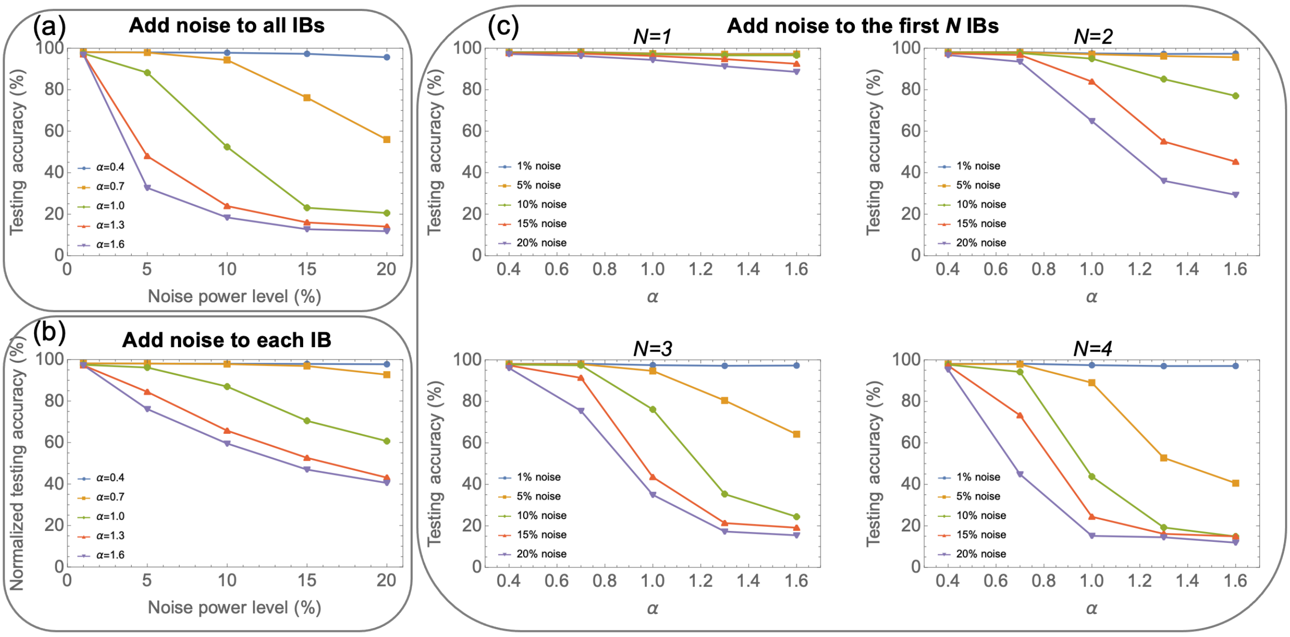

Figure S3. Testing performance of the trained CVNNs constructed with different values of α in projection layers for image classification with different noise levels.

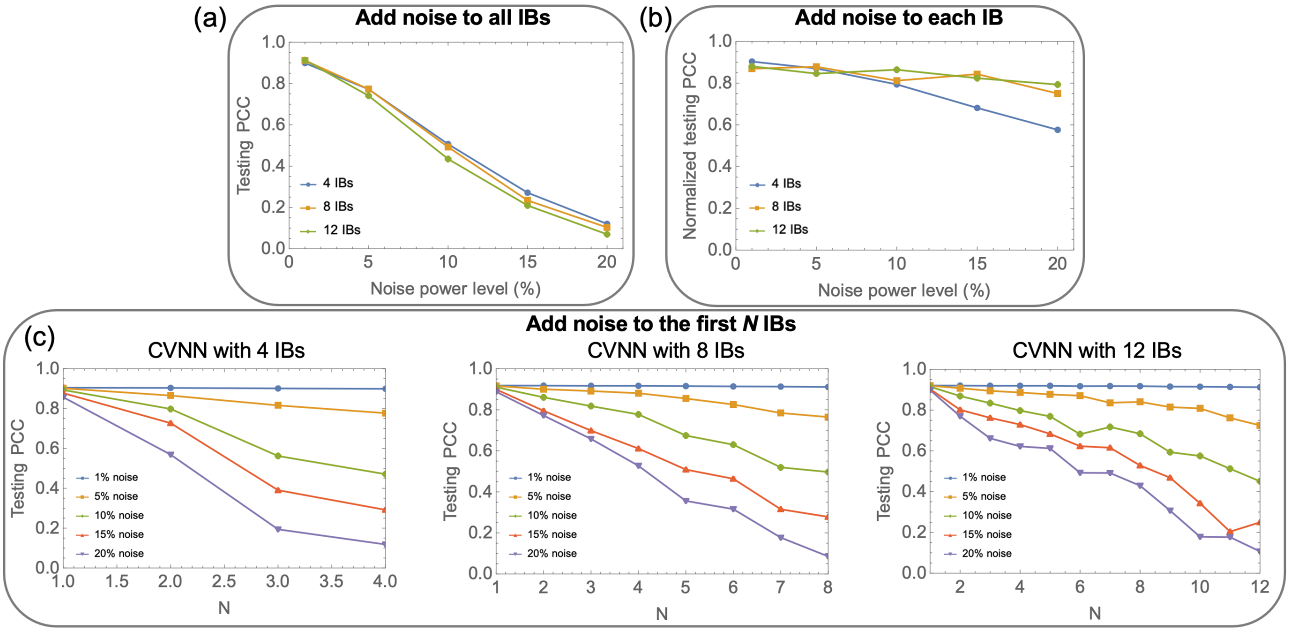

Figure S4. Testing performance of the trained CVNNs constructed with different number of IBs for image reconstruction with different noise levels.

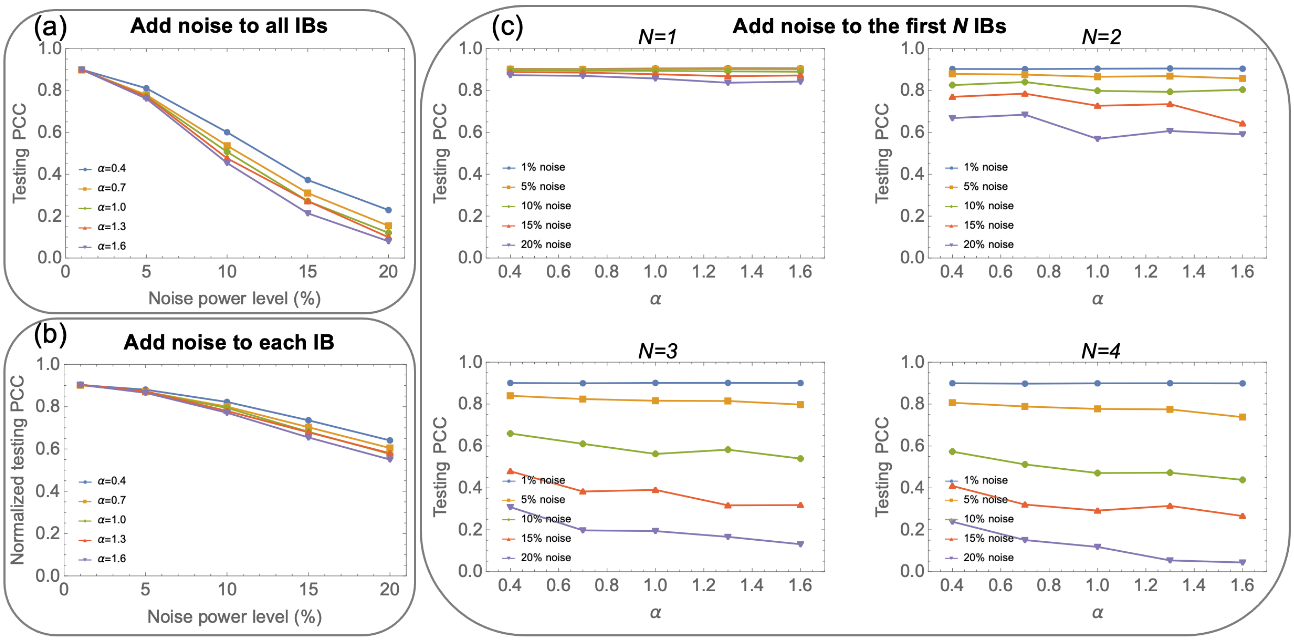

Figure S5. Testing performance of the trained CVNNs constructed with different values of α in projection layers for image reconstruction with different noise levels.

5. Governing equation of the spring-mass system

The governing equation of the spring-mass system in Figure 4(b) is described as :

$$\begin{pmatrix} m_1 & 0 & 0 & 0 & 0 \\ 0 & m_2 & 0 & 0 & 0 \\ 0 & 0 & m_3 & 0 & 0 \\ 0 & 0 & 0 & m_4 & 0 \\ 0 & 0 & 0 & 0 & m_5 \end{pmatrix} \partial_t^2 \begin{pmatrix} x_1 \\ x_2 \\ x_3 \\ x_4 \\ x_5 \end{pmatrix} + b\partial_t \begin{pmatrix} x_1 \\ x_2 \\ x_3 \\ x_4 \\ x_5 \end{pmatrix}$$

$$+ \begin{pmatrix} k_1 + k_2 & -k_2 & 0 & 0 & 0 \\ -k_2 & k_2 + k_3 & -k_3 & 0 & 0 \\ 0 & -k_3 & k_3 + k_4 & -k_4 & 0 \\ 0 & 0 & -k_4 & k_4 + k_5 & -k_5 \\ 0 & 0 & 0 & -k_5 & k_5 + k_6 \end{pmatrix} \begin{pmatrix} x_1 \\ x_2 \\ x_3 \\ x_4 \\ x_5 \end{pmatrix} = \begin{pmatrix} F_1 \\ 0 \\ 0 \\ 0 \\ 0 \end{pmatrix}. \tag{S1}$$

For simplification, we define that:

$$\boldsymbol{M} = \begin{pmatrix} m_1 & 0 & 0 & 0 & 0 \\ 0 & m_2 & 0 & 0 & 0 \\ 0 & 0 & m_3 & 0 & 0 \\ 0 & 0 & 0 & m_4 & 0 \\ 0 & 0 & 0 & 0 & m_5 \end{pmatrix},$$

$$\boldsymbol{K} = \begin{pmatrix} k_1 + k_2 & -k_2 & 0 & 0 & 0 \\ -k_2 & k_2 + k_3 & -k_3 & 0 & 0 \\ 0 & -k_3 & k_3 + k_4 & -k_4 & 0 \\ 0 & 0 & -k_4 & k_4 + k_5 & -k_5 \\ 0 & 0 & 0 & -k_5 & k_5 + k_6 \end{pmatrix}, \tag{S2}$$

$$\boldsymbol{F} = \begin{pmatrix} F_1 \\ 0 \\ 0 \\ 0 \\ 0 \end{pmatrix}.$$

With this, Eq.S1 can be rewritten as Eq.3 in the main text.

References

1. Spall, J., Guo, X., & Lvovsky, A. I. (2022). Hybrid training of optical neural networks. *Optica*, *9*(7), 803-811.
2. Fagbohungbe, O., & Qian, L. (2021, July). Benchmarking inference performance of deep learning models on analog devices. In *2021 International Joint Conference on Neural Networks (IJCNN)* (pp. 1-9). IEEE.
3. Semenova, N., Larger, L., & Brunner, D. (2022). Understanding and mitigating noise in trained deep neural networks. *Neural Networks*, *146*, 151-160.
